# Recording of HIV diagnosis in mental health records: A data linkage cohort study

**DOI:** 10.1371/journal.pone.0320392

**Published:** 2025-04-23

**Authors:** Margaret Heslin, Olivia Hunt, Emma Tassie, Amelia Jewell, Helena King, Elana Covshoff, Lucy Campbell, Sara Croxford, Rudiger Pittrof, Ann Sullivan, Julie Williams, Michael Newson, Kylee Trevillion, Shubulade Smith, Elizabeth Hughes, Robert Stewart

**Affiliations:** 1 Institute of Psychiatry, Psychology and Neuroscience, King’s College London, London, United Kingdom; 2 South London and Maudsley NHS Foundation Trust, London, United Kingdom; 3 School of Health, Wellbeing and Social Care, Open University, Milton Keynes, United Kingdom; 4 Guy’s and St Thomas NHS Foundation Trust, London, United Kingdom; 5 Faculty of Life Sciences and Medicine, School of Immunology and Microbial Science, King’s College London, London, United Kingdom; 6 Mersey and West Lancashire Teaching Hospitals NHS Trust, Prescot, United Kingdom; 7 Department of Infectious Disease Epidemiology and International Health, London School of Hygiene and Tropical Medicine, London, United Kingdom,; 8 Chelsea and Westminster Hospital NHS Foundation Trust, London, United Kingdom; 9 School of Health and Life Sciences, Glasgow Caledonian University, Glasgow, United Kingdom; Indian Institute of Information Technology, India

## Abstract

**Background:**

Mental health professionals play a crucial role in promoting the physical well-being of people with mental illness. Awareness of HIV status can enable professionals in mental health services to provide more comprehensive care. However, it remains uncertain whether mental health professionals consistently document HIV status in mental health records.

**Aims:**

To investigate the extent to which mental health professionals document previously established HIV diagnoses of people with mental illness in mental health records, and to identify the clinical and demographic factors associated with documentation or lack thereof.

**Methods:**

A retrospective cohort study was conducted using an established data linkage between routinely collected clinical data from secondary mental health services in South London, UK, and national HIV surveillance data from the UK Health Security Agency. Individuals with an HIV diagnosis prior to their last mental health service contact were included. Documented HIV diagnosis in mental health records was assessed.

**Results:**

Among the 4,032 individuals identified as living with HIV, 1,281 (31.8%) did not have their diagnosis recorded in their mental health records. Factors associated with the absence of an HIV diagnosis included being of Asian ethnicity, having certain primary mental health diagnoses including schizophrenia, being older, being with a mental health service for longer, having more clinical mental health appointments, and living in a less deprived area.

**Conclusions:**

A significant number of individuals living with HIV who are receiving mental healthcare in secondary mental health services did not have their HIV diagnosis documented in their mental health records. Addressing this gap could allow mental healthcare providers to support those living with HIV and severe mental illness to manage the complexity of comorbidities and psychosocial impacts of HIV. Mental health services should explore strategies to increase dialogue around HIV in mental health settings.

## Introduction

People with severe mental illness (schizophrenia and related disorders, bipolar disorder, depressive psychosis) have higher morbidity and mortality rates from chronic diseases compared to the general population [1,2]. This contributes to a reduced life expectancy compared to the general population of up to almost 18 years in some cases [3,4]. Increasing attention has been given to the physical health of people with severe mental illness, particularly cardiovascular and metabolic disorders, but the sexual health needs of people with severe mental illness are often neglected [5].

People with severe mental illness are at higher risk of bloodborne viruses such as HIV and hepatitis [6,7] and there is some evidence of a higher prevalence of bloodborne viruses from a systematic review and meta-analysis [5,8]. More recently, research has estimated that the prevalence of HIV in people with mental illness in the UK is 2.5 times higher than the general population [9].

HIV is treatable, and adherence to HIV medication can render the virus undetectable, meaning that it can’t be transmitted person to person, and allows individuals to live long and healthy lives [10,11]. However, there is some evidence to suggest that HIV treatment adherence is often low among people with severe mental illness [12]. For many of these individuals, mental health services are their primary, if not sole, point of contact with healthcare [13]. Consequently, mental health professionals are in a unique position to support and encourage these patients to engage with HIV treatment services [14]. Such integration could lead to a greater chance of reaching virological suppression, a lower risk of opportunistic infections and improved health and wellbeing. Moreover, certain HIV medications can have adverse interactions with psychiatric medications, underscoring the importance of mental health services being aware of their patients’ HIV status and treatment regimens [15,16]. HIV status also affects patients’ access to other healthcare services. For example, a survey of people living with HIV in Europe found that 56% of people with HIV worried about being treated differently by healthcare professionals because of their HIV status, 45% were afraid to seek services, and 36% had actively avoided healthcare services [17]. By being informed about patients’ HIV status and responding appropriately, mental health professionals can help reduce these fears and barriers, thereby encouraging engagement not only with mental health services but also with other necessary healthcare services. This holistic approach has the potential to significantly improve the overall health and wellbeing of individuals living with severe mental illness and HIV, as well as contribute to the World Health Organization target of ending HIV as a public health threat by 2030 [18].

### Aim

This paper aimed to investigate the extent to which mental health professionals document the HIV diagnosis of patients in mental health records, and to identify the clinical and demographic factors associated with the presence or absence of previously established HIV diagnoses in mental health records.

## Materials and methods

### Data sources and linkage

Data for this study come from a pre-existing data linkage reported previously [9]. Briefly, data come from a retrospective cohort study based on linked data from Electronic Health Records (EHRs) from the South London and Maudsley (SLaM) National Health Service (NHS) Trust electronic Patient Journey System (ePJS), made accessible via the Clinical Record Interactive Search tool (CRIS) at the NIHR Maudsley Biomedical Research Centre (BRC), and comprehensive national HIV surveillance data held at the UK Health Security Agency (UKHSA), formerly Public Health England (PHE). CRIS hold mental health records from over 500,000 patients in SLaM who provides near-monopoly secondary mental healthcare to the four London boroughs of Lambeth, Southwark, Lewisham and Croydon (approximately 1.3m residents). SLaM provides a wide range of NHS mental health services including mental health and substance use services.

Data were linked between the two data sources for all adults (aged ≥ 16 years) who had a contact with secondary mental health services for the first time between 1^st^ January 2007 and 31^st^ December 2018 (HIV data extracted 19^th^ of November 2020). A hierarchical matching algorithm was devised to match the datasets (see [9] for further details). Confirmed HIV diagnosis and other clinical HIV data were returned to CRIS for research purposes.

### Sample

For this study, all individuals matched during the linkage process were eligible to be included in the sample. However, individuals with an HIV diagnosis date that occurred after their last contact with mental health services were excluded.

### Measures

**Fig 1:**
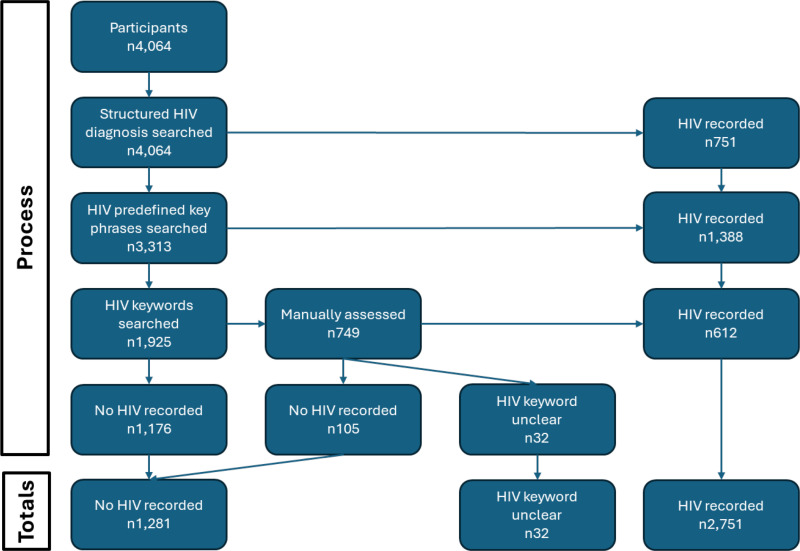
Flow diagram of process to determine presence/absence of HIV diagnosis in mental health records.

The main outcome measure included in these analyses was the presence or absence of recorded HIV diagnoses in mental health records, categorized as a binary variable: HIV recorded versus no HIV recorded. The mental health records searched included all free text notes from events, and all forms and attachments including correspondence to and from the service, assessments, care plans, referrals and discharge plans/letters. To create this variable, a systematic process was followed, as shown in Fig 1.

1. Structured Clinical Diagnoses: The first step involved examining structured clinical diagnoses for specific ICD codes indicative of an HIV diagnosis: B20 * , B21 * , B22 * , B23 * , B24 * , Z21 * , R75 * , Z71.7 * , and F02.4 * . If any of these codes were present, the patient was recorded as having a confirmed HIV diagnosis.
2. Key Phrase Search: For records without these ICD codes, the next step was to search patient records (including clinical notes (e.g., events, notes, attachments etc) and forms (e.g., risk assessments)) using a list of predefined key phrases. These phrases, listed in Supplement 1, were piloted to ensure sensitivity and specificity. The presence of any key phrase indicated a recorded HIV diagnosis.
3. Keyword “HIV” Search: If neither ICD codes nor key phrases were found, the records were then searched (including clinical notes and forms as above) for the keyword “HIV.” Any instances of this keyword were manually assessed by two independent raters (OH and ET) to determine if they confirmed an HIV diagnosis. Disagreements between the two raters were resolved by a third rater (MH).

Overt statements of HIV diagnosis were included, but indications of HIV status, even if not explicitly stated, were also included. For example, both “Currently HIV+” and more indirect references like “She found it difficult to discuss HIV with staff” or “Refer to HIV liaison” were included. Additionally, any communication between the mental health service and an HIV service was considered a confirmed HIV diagnosis in the records.

Following this process, additional searches were performed for those who had no HIV recorded. This included checking lab results related to HIV, and searching for the terms “viral load”, “ARV” and “retroviral” to make sure HIV had not been referenced in another way. Additionally, key HIV services in the area were searched for to check if these services were referenced in the notes despite no “HIV”.

HIV diagnosis dates were extracted from the national HIV surveillance data. All other data were extracted from CRIS. Ethnicity, date of birth (later transformed into age at first contact with mental health services), and date of last contact with mental health services during the study window were extracted for each person. Number of months care of mental health service in study window, number of mental health inpatient bed days in the study window, the number of clinical mental health appointments with mental health services (face-to-face, video and phone call) in study window (divided by 10 to aid statistical interpretation) were all extracted from the data set. The total number of distinct mental health symptoms in the study window were extracted using NLP apps [19]. Mental health diagnosis was defined as the most recent primary mental health diagnosis from structured data and extracted. Diagnoses were categorised into the ICD-10 mental and behavioural disorder categories (ICD-10) [20] plus a category for no formal mental health diagnosis. Post code for the active address nearest the time of HIV diagnosis (before or after) was linked to Lower Super Output Areas (LSOA) and extracted. This was linked to 2019 English indices of deprivation [21] and the Index of Multiple Deprivation decile was used (where a rank of 1 indicates the most deprived area).

All variables were measured during the study window which was 01/01/2007-20/09/2022 (date of data extraction). Additional data extracts were conducted up to 01/10/2024.

### Analysis

Having described recorded HIV diagnosis, differences between those with and without a recorded HIV diagnosis were described and then analysed using logistic regression. A bootstrap logistic regression was used as some variables were skewed, and bootstrapping is a robust method for handling non-normally distributed data [22] Variables identified as being statistically significant in the univariate analysis (p < 0.05) were then simultaneously included in a multivariate analysis. Data analysis was conducted using complete case analysis, and no additional analysis for missing data was performed. All analyses were conducted using STATA Version 18 [23]. Multicollinearity was assessed using a correlation matrix and variance inflation factor.

Substance use disorders were used as the reference group in diagnosis regressions as substance use teams are trained to be aware of, and advise on etc bloodborne viruses in their populations.

### Ethical approval

Ethical approval was granted by the South Central - Oxford C Research Ethics Committee (REC reference: 23/SC/0257). Section 251 of the NHS Act 2006 by the Health Research Authority (HRA) Confidentiality Advisory Group (CAG) (CAG reference: 19/CAG/0127) was granted. No informed consent was taken to access data in line with ethics approval for this study, but any SLaM patient that registered a local or national opt-out were excluded. The technical team had access to identifiable information for the original linkage, however, all data were de-identified for data extraction and analysis.

## Results

### Sample

A total of 4,481 individuals in contact with mental health services in SLaM during the study period had a recorded HIV diagnosis in national HIV surveillance data at any time. Of those, 417 (9%) had their HIV diagnosis after their last contact with mental health services and were thus excluded from these analyses. Therefore, a total of 4,064 individuals were included.

Fig 1 shows the process followed to determine presence or absence of an HIV diagnosis in the mental health records. Of the 4,064 included in the sample, 751 (18.5%) had an HIV-related diagnosis in structured diagnosis fields. A further 1,388 (34.2%) had an HIV predefined key phrase recorded in their mental health records. A total of 749 (18.4%) had HIV as a keyword in their mental health records; however, this was only manually confirmed as a recorded HIV diagnosis in 612 of those cases (15.1% of the full sample). Thirty-two (<0.1%) could not be manually classified due to ambiguity in the text, and 105 (<0.1% of the full sample) were manually confirmed to not include a recorded HIV diagnosis. Finally, 1,176 (28.9%) had no HIV-related diagnosis recorded in structured diagnosis fields, HIV predefined key phrase, or HIV keyword anywhere in the mental health records.

Interrater agreement on manually assessed records was 86.4% (647/749); however, all differences were resolved by a third rater.

Overall, excluding individuals who could not be classified, 68.2% (2,751/4,032) of individuals had their HIV diagnosis documented in their mental health records, while 31.8% (1,281/4,032) did not. Among the 1,281 individuals without a recorded HIV diagnosis, none had a confirmed HIV test in their lab results, and none had reference to “viral load” or “retroviral” in relation to HIV. However, 70 individuals in this group had reference to either “ARV” or a specific HIV service noted in their records. These 70 individuals were not included in the analysis below as the focus was on documented HIV diagnoses, rather than indirect references that would require specialist knowledge to interpret this accurately.

### Descriptives

Table 1 describes the characteristics of the people included in the sample. The majority (74.8%) were male. The mean age at first contact with mental health services was 40.3 years (10.8 years SD) with a range of 16-90 years old. The most common ethnicity was White British (33.0%), followed by Black African (13.9%) and White Other (12.8%). An unknown or missing ethnicity was recorded for 18.2% of the sample. In terms of most recent primary mental health diagnosis, the largest diagnostic group was substance disorders (17.8%), followed by mood disorders (16.5%), and neurotic, stress-related and somatoform disorders (14.8%). An unknown or missing diagnosis was recorded for 32.1% of the sample.

**Table 1:** Sample demographics.

	Included sample (4,032)
	N (%)
**Gender (missing n = 2)**	
Male	3,013 (74.8)
Female	1,017 (25.2)
**Ethnicity (missing = 0)**	
White British	1,331 (33.0)
White Other	516 (12.8)
Mixed Ethnicity	72 (1.8)
Asian	102 (2.5)
Black Caribbean	135 (3.4)
Black African	562 (13.9)
Black Other	252 (6.3)
Other	327 (8.1)
Unknown	735 (18.2)
**Primary mental health diagnosis (missing = 0)**	
F0: organic disorders	291 (7.2)
F1: substance disorders	718 (17.8)
F2: schizophrenia and related	186 (4.6)
F3: mood disorders	666 (16.5)
F4: neurotic, stress-related and somatoform disorders	595 (14.8)
F5: behavioural syndromes associated with physiological disturbances and physical factors	84 (2.1)
F6: disorders of adult personality and behaviour	112 (2.8)
F7: intellectual disability	13 (0.3)
F8: disorders of psychological development	10 (0.3)
F9: behavioural and emotional disorders with onset usually occurring in childhood and adolescence	61 (1.5)
No mental health diagnosis	1,296 (32.1)
	**Mean (SD), range**
**Age at first contact with mental health service (missing n = 86)**	40.3 (10.8), 16.0-89.9

### Associations between clinical/demographic factors and presence of HIV diagnosis

Table 2 displays demographic and clinical characteristics of the sample according to the presence or absence of a recorded HIV diagnosis, plus univariate odds ratios for having an HIV diagnosis recorded in mental health records. An odds ratio greater than 1 indicates that the group in question was more likely to have HIV recorded in the notes compared to the reference group. Conversely, an odds ratio less than 1 indicates that the group is less likely to have HIV recorded in the notes compared to the reference group.

**Table 2:** Demographic and clinical factors associations with recorded HIV diagnosis.

	Recorded HIV diagnosis (2,751)	No recorded HIV diagnosis (1,281)	Odds ratio of the HIV diagnosis being recorded
	N (%)	N (%)	OR, 95%, p value
**Gender (missing n = 2)**			
Male	2,061 (68.4)	952 (31.6)	–
Female	689 (67.8)	328 (32.3)	0.97, 0.84-1.12, 0.683
**Ethnicity (missing = 0)**			
White British	834 (62.7)	497 (37.3)	–
White Other	420 (81.4)	96 (18.6)	2.61, 2.04-3.34, < 0.001
Mixed Ethnicity	54 (75.0)	18 (25.0)	1.79, 0.99-3.20, 0.051
Asian	47 (46.1)	55 (53.9)	0.51, 0.34-0.80, 0.001
Black Caribbean	92 (68.2)	43 (31.9)	1.27, 0.86-1.88, 0.2222
Black African	485 (86.3)	77 (13.7)	3.75, 2.90-4.86, < 0.001
Black Other	181 (71.8)	71 (28.2)	1.52, 1.12-2.05, 0.007
Other	217 (66.4)	110 (33.4)	1.18, 0.91-1.52, 0.214
Unknown	421 (57.3)	314 (42.7)	0.80, 0.66-0.96, 0.017
**Primary mental health diagnosis (missing = 0)**			
F0: organic disorders	238 (81.8)	53 (18.2)	1.81, 1.29-2.53, 0.001
F1: substance disorders	512 (71.3)	206 (28.7)	–
F2: schizophrenia and related	111 (59.7)	75 (40.3)	0.60, 0.42-0.84, 0.003
F3: mood disorders	480 (72.1)	186 (27.9)	1.04, 0.82-1.31, 0.749
F4: neurotic, stress-related and somatoform disorders	445 (74.8)	150 (25.2)	1.19, 0.93-1.53, 0.167
F5: behavioural syndromes associated with physiological disturbances and physical factors	46 (54.8)	38 (45.2)	0.49, 0.30-0.78, 0.003
F6: disorders of adult personality and behaviour	89 (79.5)	23 (20.5)	1.56, 0.95-2.55, 0.079
F7: intellectual disability	3 (23.1)	10 (76.9)	0.12, 0.03-0.45, 0.002
F8: disorders of psychological development	4 (40.0)	6 (60.0)	0.27, 0.06-1.11, 0.069
F9: behavioural and emotional disorders with onset usually occurring in childhood and adolescence	29 (47.5)	32 (52.5)	0.36, 0.21-0.63, < 0.001
No mental health diagnosis	794 (61.3)	502 (38.7)	0.64, 0.52-0.78, < 0.001
**Mental health inpatient stay during period (missing = 0)**			
No	2,361 (66.6)	1,183 (33.4)	–
Yes	390 (79.9)	98 (20.1)	1.99, 1.57-2.53, < 0.001
	**Mean (SD)**	**Mean (SD)**	**OR, 95%, p value**
**Age at first contact with mental health service (missing n = 86)**	39.84 (10.6)	41.29 (11.2)	0.99, 0.98-0.99, < 0.001
**Index of multiple deprivation decile (missing = 267)**	3.76 (1.72)	4.30 (2.18)	0.86, 0.83-0.90, < 0.001
**Number of months under care of mental health service in study window (missing = 0)**	17.55 (23.98)	15.03 (26.06)	1.00, 1.00-1.01, 0.007
**Number of mental health inpatient days in study window (missing = 0)**	8.40 (50.70)	10.23 (84.89)	<1.00, < 1.00-1.00, 0.483
**Number of clinical mental health appointments in study window/10 (missing = 0)**	1.98 (4.06)	1.48 (4.34)	1.03, 1.01-1.06, 0.008
**Total number of distinct mental health symptoms in study window (missing = 0)**	10.14 (8.36)	6.68 (7.97)	1.06, 1.05-1.07, < 0.001

In terms of ethnicity, compared to individuals identified as White British, those identified as White Other, Black African, and Black Other were more likely to have an HIV diagnosis recorded in their records. Conversely, individuals identified as Asian or with an unknown ethnicity were less likely to have an HIV diagnosis recorded.

In terms of primary mental health diagnosis, compared to substance use disorders, a diagnosis of organic disorder was associated with a higher likelihood of having an HIV diagnosis being recorded. Conversely, diagnoses of schizophrenia and related disorders, behavioural syndromes associated with physiological disturbances and physical factors, intellectual disability, behavioural and emotional disorders with onset usually occurring in childhood and adolescence, as well as the absence of a recorded mental health diagnosis, were all associated with a lower likelihood of having HIV recorded in mental health records.

Having a higher age at first contact with mental health services was associated with not having HIV recorded in the mental health records as was living in an area with a higher index of multiple deprivation decile – meaning lower deprivation (higher decile indicates less deprivation). There were no differences observed by gender.

Higher contact with services (months under care of service, clinical mental health appointments) was associated with an HIV diagnosis being recorded as was having a higher number of distinct mental health symptoms. Although ever being an inpatient was associated with HIV being recorded, there was no association in recording HIV and number of inpatient days itself.

Significant variables identified in the univariable analysis were simultaneously included in a single multivariate analysis (Table 3). Results indicated that older age, living in areas with less deprivation (as higher deciles indicate lower deprivation), and a higher number of months under the care of mental health services were associated with an individual not having an HIV diagnosis documented in their mental health records. Conversely, a higher number of distinct mental health symptoms remained associated with having an HIV diagnosis recorded. Of note, the direction of association for the number of clinical mental health appointments shifted after adjustment; in the univariable analysis, fewer clinical mental health appointments were linked to a lower likelihood of having an HIV diagnosis recorded, but in the multivariate analysis, more clinical mental health appointments were associated with a lower likelihood of having the diagnosis recorded.

**Table 3:** Multivariate analysis of demographic and clinical factors associations with recorded HIV diagnosis.

	Odds ratio of the HIV diagnosis being recorded
	OR, 95%, p value
**Ethnicity**	
White British	–
White Other	2.51, 1.88-3.33, < 0.001
Mixed Ethnicity	1.40, 0.69-2.84, 0.346
Asian	0.47, 0.30-0.76, 0.002
Black Caribbean	1.16, 0.77-1.74, 0.491
Black African	3.43, 2.57-4.59, < 0.001
Black Other	1.38, 1.00-1.91, 0.051
Other	1.07, 0.81-1.40, 0.646
Unknown	1.08, 0.87-1.35, 0.468
**Primary mental health diagnosis**	
F0: organic disorders	1.77, 1.19-2.65, 0.005
F1: substance disorders	–
F2: schizophrenia and related	0.27, 0.17-0.41, < 0.001
F3: mood disorders	0.75, 0.56-0.99, 0.044
F4: neurotic, stress-related and somatoform disorders	1.05, 0.81-1.38, 0.696
F5: behavioural syndromes associated with physiological disturbances and physical factors	0.77, 0.48-1.23, 0.272
F6: disorders of adult personality and behaviour	1.18, 0.67-2.08, 0.573
F7: intellectual disability	0.08, 0.02-0.34, 0.001
F8: disorders of psychological development	0.26, 0.06-1.18, 0.081
F9: behavioural and emotional disorders with onset usually occurring in childhood and adolescence	0.37, 0.19-0.69, 0.002
No mental health diagnosis	0.83, 0.66-1.06, 0.133
**Mental health inpatient stay during period**	
No	–
Yes	0.87, 0.62-1.22, 0.409
	**OR, 95%, p value**
**Age at first contact with mental health service**	0.99, 0.98- < 1.00, 0.003
**Index of multiple deprivation decile**	0.87, 0.84-0.91, < 0.001
**Number of months under care of mental health service in study window**	0.99, 0.99- < 1.00, 0.010
**Number of clinical mental health appointments in study window/ 10**	0.96, 0.93-0.99, 0.010
**Total number of distinct mental health symptoms in study window**	1.09, 1.07-1.11, < 0.001

Regarding ethnicity, individuals who identified as White Other, and Black African continued to show increased odds of having an HIV diagnosis recorded, whereas those identified as Asian had decreased odds. In terms of diagnosis, having an organic disorder was associated with a higher likelihood of an HIV diagnosis being recorded. In contrast, diagnoses of schizophrenia, intellectual disability, or behavioural and emotional disorders typically arising in childhood and adolescence were linked to a lower likelihood of an HIV diagnosis being recorded. Additionally, having a mood disorder was associated with a decreased likelihood of having an HIV diagnosis recorded in the multivariate analysis. In this multivariate analysis, multicollinearity was deemed not to be an issue (maximum correlation =  0.7108; mean variance inflation factor =  1.60).

## Discussion

### Principal findings

This study found that almost a third (31.8%) of people who were living with HIV and were in contact with mental health services did not have their HIV diagnosis documented in their mental health records. Awareness of a patient’s HIV diagnosis is crucial for mental health professionals for several reasons. This awareness can be used to identify potential contraindications between HIV and mental health medications, allow clinicians to ensure that possible organic causes of mental health symptoms are diagnosed in a timely fashion, allow clinicians to support patients to manage the complexity of comorbidities and psychosocial impacts of HIV, and if necessary, enable clinicians to encourage and support patients in accessing HIV care and treatment services. Additionally, awareness of a patient’s HIV status allows mental health professionals to monitor for early signs of physical health complications and to provide timely referrals and signposting.

The finding that almost a third of people who were living with HIV and were in contact with mental health services did not have their HIV diagnosis documented in their mental health records is surprisingly high. This is especially surprising in light of the fact that bloodborne testing on admission to inpatient units is now standard across the trust that this study was conducted in. However, this standard of bloodborne testing is a relatively recent addition, and only 12% of the sample were treated as inpatients, the majority were treated as outpatients. Additionally, mental health staff are able to access patient’s Summary Care Record which should have HIV status and associated medications recorded. However, this is a relatively recent change and even if staff are accessing this, they are not recording the information in the mental health records which was the focus of this study.

There was a distinct difference between the recording of HIV status for people with substance use disorders as the primary mental health diagnosis compared with those without a primary substance use disorder. This is likely due to the fact that an assessment of risk of bloodborne virus infections is a part of routine assessment in substance use treatment [24], whereas this is not the case in mental health services. This likely means that patients were not asked about their HIV status. One solution to this is to make bloodborne virus enquiry a routine as part of mental health assessment and perhaps annual physical health checks. This would mean that each patient would be asked at initial assessment, and at regular intervals while still in contact with services. However, this raises issues around competence and stigma in mental health professional staff. Hendry et al [25] found that some mental health nurses avoid sexual health discussions due to their own discomfort and/or personal beliefs around sex and sexuality, worry about being accused of misconduct, dehumanisation of those with mental health issues, and lack of education on the topic. Clearly, these issues need rectifying before routine enquiry around sexual health could begin.

A further issue which needs to be addressed is disclosure. Even if every patient was asked about HIV status, some may likely not disclose. As outlined above, over half of people with HIV worried about being treated differently by healthcare professionals because of their HIV status [17], which is likely to impact disclosure. One approach to improving disclosure could be for mental healthcare providers to sign up to the HIV Confident Charter [26] or similar programmes. The HIV Confident Charter is a partnership between National AIDS Trust, Aidsmap and Positively UK, supported by Fast Track Cities London. It aims to ensure that people living with HIV can access services and employment without fear of discrimination by committing to increasing employee knowledge about HIV, improving attitudes towards people living with HIV, tackling stigma and discrimination within organizations, and providing ways to report any stigma or discrimination experienced [26]. Alternatively, healthcare providers should adhere to British HIV Association standards of care [27]. These state that staff across all health and social care services, including mental health, should receive basic information and training on bloodborne viruses and HIV related stigma, and that people living with HIV should be made aware of how they can raise concerns if they are unhappy with their care or have experienced stigma. In theory, these approaches could improve the mental health experience of people with HIV and increase their willingness to disclose their HIV status.

In terms of the search terms that were included, PEP or PrEP were excluded as this is used prophylactically and does not indicate a confirmed diagnosis. Additionally, the names of specific HIV medications were excluded for two reasons. First, these medications might have been prescribed as PEP or PrEP. Additionally, referencing medication names without clarifying that they are HIV-related assumes a level of HIV knowledge that many mental health clinicians likely do not possess, given the generally low levels of HIV literacy among mental health staff [28].

There is evidence that mental health professionals are becoming more aware of the importance of knowing about the physical health of their patients, and ensuring they receive the appropriate treatment. However, it has been reported that they often have a lack of confidence in how to address physical health issues and concern about their lack of knowledge [29, 30]. This is true of all physical health conditions and may be more of an issue with HIV where there may also be a lack of knowledge which may be exacerbated by stigma [31]. There is little evidence on how to support mental health professionals in having discussions with patients about their HIV status but increasing knowledge and decreasing stigma individually, alongside changing culture at team and organisation level may help this. Providing mental health professionals with training on sexual health, bloodborne viruses and HIV, as well as equipping them with skills to ask questions about HIV and sexual health in a sensitive and non-judgemental manner, may improve the situation. However, education alone is often insufficient to lead to behaviour change. Therefore, any new training on this topic should be evaluated for changes in knowledge, stigma and integration of routine enquiry into clinical practice.

In terms of generalisability, this study was based on data from a single NHS trust in South London, UK which may limit the broader applicability of the findings. Therefore, caution is needed when extrapolating these results to the wider UK or global populations. However, it is important to note that two of the four London boroughs within the trust’s catchment area had the highest HIV prevalence rates in the country at the time of data collation [32]. As a result, awareness of HIV in this trust may be higher, and mental health services in these areas may be particularly alert to HIV-related concerns in their patient populations. Consequently, it is possible that documentation of HIV in mental health records may be lower in regions where HIV prevalence is lower.

The association between clinical and demographic variables, and the presence of an HIV diagnosis was explored using logistic regression models. However, the r-squared for the final multivariate analysis was 0.1039, indicating that only 10% of the variance in whether HIV diagnosis was recorded in mental health records was explained by the variables included in the model. This relatively low value suggests that important predictors of HIV diagnosis recording in mental health records were not measured in this study.

### Strengths and limitations

This study has a number of strengths. Firstly, it is the first study to explore whether confirmed HIV status is documented in the mental health records of people in contact with mental health services. The use of linked data on HIV diagnosis from the UK Health Security Agency (UKHSA) is a strength, as this national dataset captures diagnoses and treatments across the UK. A further strength is the use of double coding for HIV status in manually assessed records, with a third coder resolving any disagreements.

However, there are some limitations that need to be considered. First, there is an implicit assumption that if HIV was not recorded in the mental health records, the mental health professionals must have been unaware of the HIV diagnosis. However, it is possible that some professionals were aware but did not document this. It is also possible that HIV status would have been included in handwritten referral letters and were not included as such here. Second, clinical data can be difficult for non-clinical individuals to interpret due to the use of shorthand [33] and this study was led by academics, not clinicians. Additionally, this data came from a single UK site. It is possible that other service providers include routine enquiry about HIV, or document HIV more consistently than this site. However, between-clinician differences are more likely than between-service differences, and this study was based on a very large provider providing support for approximately 1.3 million people in South London. In terms of analysis, this was conducted using complete case analysis, excluding those with missing data. While this approach could introduce bias, as those with missing data could differ significantly different to those without missing data, the dependant variable was available for the entire cohort as records were accessible for all. Additionally, the independent variables were nearly all complete, with those having missing data showing a minimum of 82% completion.

In this study, HIV recording may have been overestimated, as even a single mention of HIV was categorized as a recorded case. However, in clinical practice, recording something as critical as an HIV diagnosis only once does not guarantee continuity of care. If an HIV diagnosis is known, it should be referenced in every formal communication between healthcare providers. Instances where HIV is mentioned only once or twice may indicate inadequate documentation for ensuring continuity of care. Moreover, excluding cases with unclear mentions of HIV likely inflates reporting, as these do not provide definitive information to the reader.

Finally, using secondary data means certain information may not have been available [34]. For example, it would have been useful to explore other factors associated with the presence or absence of HIV diagnoses in mental health records, such as migration status, where the person received their HIV diagnosis, and the reason for contact with mental health services.

## Conclusions

A significant number of individuals using mental health services and living with HIV did not have their HIV diagnosis documented in their mental health records. People with substance use disorders were more likely to have HIV status recorded than those with other mental health diagnoses which reflects the inclusion of bloodborne virus checks in clinical guidelines for Drug Misuse and Dependence. Given that people using mental health services are more at risk of HIV (and other bloodborne viruses) infections, mental health services need to urgently address how they assess for risk of bloodborne viruses as well as establish HIV status. This will ensure that people get access to timely treatment which will not only enhance their own health and well-being but play a significant part in reducing transmission and ultimately elimination of HIV.

## Supporting information

S1 File Key HIV confirmation terms. (DOCX)
