# Supplementary material for: Recording of HIV diagnosis in mental health records: A data linkage cohort study
Source: PLoS One. 2025 Apr 23;20(4):e0320392. doi: 10.1371/journal.pone.0320392 (PMC12017511; doi:10.1371/journal.pone.0320392)
Supplement: S1 File — (DOCX) [file pone.0320392.s001.docx]

**Supplement**

**Supplement 1 – Key HIV confirmation terms**

| HIV liaison |
| --- |
| HIV concerns |
| background HIV |
| PMH: HIV |
| PMH HIV |
| PM Hx: HIV |
| PMHx: HIV |
| PMHx HIV |
| PM Hx HIV |
| HIV team |
| HIV services |
| CD4 |
| Medical history HIV |
| Medical history: HIV |
| HIV medication |
| HAART |
| Antiretroviral |
